# Effects of smoking on the optimal effect-site concentration of remifentanil required for preventing cough during anesthetic emergence in male patients undergoing laparoscopic or robotic cholecystectomy

**DOI:** 10.1097/MD.0000000000025288

**Published:** 2021-04-02

**Authors:** Ha Yeon Kim, Jong Bum Choi, Eunyoung A. Lee, Sei Hyuk Kwon, Ji Eun Kim, Sook Young Lee

**Affiliations:** a Department of Anesthesiology and Pain Medicine; b Department of Biomedical Informatics, Ajou University School of Medicine, 164, World Cup-Ro, Yeongtong-Gu, Suwon 16499, Republic of Korea.

**Keywords:** cough, effect-site concentration, remifentanil, smoking

## Abstract

Target-controlled infusion of remifentanil is known to reduce cough effectively during emergence from general anesthesia. The effect of smoking on emergence cough remains controversial. Therefore, we aimed to investigate the effect-site concentration (Ce) of remifentanil in the male patients undergoing laparoscopic or robotic cholecystectomy for suppressing emergence cough in smokers and non-smokers.

Twenty smokers and 24 non-smokers (sex, male; age range, 20–65 years) were enrolled in this study. Anesthesia was maintained using sevoflurane and remifentanil. The Ce of remifentanil in 50% (EC_50_) and 95% (EC_95_) of the patients required for suppressing emergence cough were determined for each group (smokers and non-smokers) using Dixon up-and-down method and isotonic regression method with a bootstrapping approach.

Dixon up-and-down method revealed that the EC_50_ value was significantly higher in smokers (3.51 ± 0.60 ng/mL) than in non-smokers (2.71 ± 0.30 ng/mL) (*P* < 0.001). In smokers and non-smokers, isotonic regression revealed EC_50_ to be 4.40 (83% CI, 4.17–4.58) ng/mL and 2.58 (83% CI, 2.31–2.87) ng/mL, respectively, and EC_95_ to be 4.76 (95% CI, 4.73–4.78) ng/mL and 3.15 (95% CI, 3.04–3.18) ng/mL, respectively.

The Ces of remifentanil required to prevent cough during emergence were significantly higher in smokers than in non-smokers. Therefore, clinicians should pay attention to the smoking history of a patient to prevent cough during emergence.

## 1 Introduction

Cough suppression during emergence from general anesthesia has become a crucial issue concerning patient safety. Cough during emergence may lead to inadvertent effects such as laryngospasm, hypertension, tachycardia, and increased intracranial and intraocular pressures.^[1]^

Remifentanil is a potent ultrashort-acting opioid, with rapid blood/effect-site equilibrium and elimination.^[2]^ Remifentanil is rapidly eliminated by non-specific blood and tissue esterases, without accumulation, even after prolonged infusion during surgery. In addition, low-dose infusion of remifentanil is known to decrease cough during emergence.^[3,4]^ To customize the infusion rate of remifentanil for individual patients and to achieve the desired target concentration, target-controlled infusion (TCI) has been used. So far, numerous studies have investigated the optimal effect-site concentration (Ce) of remifentanil for the prevention of cough during emergence and extubation_._^[5–13]^

The effect of a patient's smoking history on cough during emergence remains controversial.^[14–16]^ In addition, there are no reports on studies that compared the optimal Ce of remifentanil between smokers and non-smokers. The aim of this study was to investigate the optimal Ce of remifentanil for 50% of patients (EC_50_) and that for 95% of patients (EC_95_) required for the prevention of cough during anesthetic emergence and to compare the difference in Ce according to smoking history in male patients undergoing laparoscopic or robotic cholecystectomy.

## 2 Methods

### 2.1 Study population

This study was approved by the Institutional Review Board of Ajou University Hospital (AJIRB-MED-OBS-17–107, December 6, 2017) and registered at http://cris.nih.go.kr (KCT0003102). Written informed consent was obtained from all participants. Male patients (age range, 20–65 years) with a physical status score of 1 or 2 according to the American Society of Anesthesiologists (ASA) classification system, who were scheduled to undergo laparoscopic or robotic cholecystectomy, were enrolled from May 2018 to September 2018. Patients who had an upper respiratory-tract infection 2 weeks before surgery, a body mass index of more than 30 kg/m^2^, and history of difficult intubation, asthma, chronic obstructive pulmonary disease, uncontrolled hypertension or diabetes mellitus, or more than a moderate degree of coronary artery disease and those who belonged to class 3 or 4 according to Mallampati classification were excluded.

### 2.2 Anesthesia

All the patients were administered anesthesia without pre-medication. In the operating room, the patient was subjected to basic monitoring, which included pulse oximetry, electrocardiography, noninvasive arterial pressure measurement, end-tidal carbon dioxide (EtCO_2_), and Anesthetic Depth Monitor for Sedation (ADMS^TM^, Unimedics CO., LTD., Seoul, Korea). Anesthesia was induced with intravenous (IV) propofol (1.5–2.5 mg/kg) and a target concentration (1–5 ng/mL) of remifentanil (Tivare; BCWORLD PHARM. CO., LTD., Seoul, Korea) using TCI mode and a commercial anesthesia infusion pump (Orchestra Base Primea; Fresenius Vial, Brezins, France); Minto pharmacokinetic model was used for TCI.^[17]^ After the loss of consciousness, 0.8 mg/kg of IV rocuronium was administered. Oral tracheal intubation was performed using an endotracheal tube, which has an internal diameter of 8.0 mm. Cuff pressure was adjusted, such that it was maintained between 20 and 25 mmHg, using a hand pressure gauge.

Anesthesia was maintained by administering sevoflurane (1.5–2.5 vol%) and remifentanil (Ce of 1–5 ng/mL). Anesthetic depth was controlled by maintaining a unicon index (uCON) of 40–60 and a baseline heart rate (HR) and mean arterial pressure (MAP) with no more than 20% fluctuation. Mechanical ventilation was maintained with an air/oxygen mixture; a FiO_2_ (fraction of inspired oxygen) of 0.5 L/min, a gas flow of 3 L/min, and an EtCO_2_ of 35–40 mmHg were targeted.

When skin suturing began, sevoflurane dose was adjusted to maintain an approximate uCON level of 60; the predetermined Ce was maintained. For postoperative pain and postoperative nausea and vomiting (PONV) control, 30 mg of IV ketorolac and 0.3 mg of ramosetron were administered. At the end of the surgery, sevoflurane was withdrawn and fresh gas flow was increased to 10 L/min. Oral suction was gently performed, and 3 mg/kg of sugammadex was injected to reverse a neuromuscular block, which was checked as more than 90% response of train-of-four ratio. Mechanical ventilation was then switched with manual ventilation, and EtCO_2_ was maintained at 35–40 mmHg. After the patients opened their eyes in response to verbal commands without other stimulation and after adequate spontaneous ventilation was confirmed, the endotracheal tube was removed. Immediately after extubation, remifentanil infusion was stopped, and 100% oxygen was supplied using a facial mask. After observation for 5 minutes, the patients were transferred to post-anesthesia care unit (PACU).

### 2.3 Study protocol and outcome measurements

Data on preoperative demographics, smoking history, and past medical history were collected. The number of times intubation was attempted and endotracheal tube was repositioned was recorded during the induction of anesthesia. HR, MAP, and saturation via peripheral pulse oximetry were recorded at 5-time points: baseline (before induction), the end of surgery, immediately before and after extubation, and 5 min after extubation.

Patients were divided into two groups according to their smoking history: smoker group and non-smoker group. If a patient had a smoking history of at least 5 cigarettes a day for more than 6 months in the period before surgery, the patient was considered as a smoker.^[14]^ The patients were enrolled according to Dixon up-and-down sequential allocation. Cough was defined as the strong and sudden contraction of abdominal muscle. Cough during emergence was recorded from the time of the end of surgery to 5 min after extubation. In the first patient of each group, the Ce of remifentanil was 2 ng/mL. In the next patient, the Ce of remifentanil was determined on the basis of the cough response of the previous patient. If the patient did not cough during emergence, it was defined as smooth extubation (success), and the Ce for the next patient was reduced by 0.4 ng/mL. If the patient coughed more than once during emergence, it was defined as failed smooth extubation (failure), and the Ce of the next patient was increased by 0.4 ng/mL.

Hemodynamic data, which included HR and MAP during the perioperative period, were recorded at 5-time points: before induction, at the end of operation, immediately before and after extubation, and 5 min after extubation. When a patient opened his eyes in response to a verbal command, the end-tidal concentration of sevoflurane was recorded. The time to extubation was defined as the period from the time of sevoflurane withdrawal to that of extubation. Complications, such as bradypnea with a respiratory rate of <8 bpm, laryngospasm, and saturation <95% despite oxygen supplementation, were recorded 5 min after extubation. In the PACU, sedation was scored using the Ramsay Sedation Scale (6 levels: 1, anxious and agitated or restless or both; 6, no response to a light glabellar tap or loud auditory stimulus)^[18]^ and pain was scored using an 11-point numerical rating scale (NRS; 0, no pain; 10, worst pain). Fentanyl (0.5 mcg/kg) was administered if the NRS score was 5 or higher. A patient was considered to have PONV when the patient experienced nausea that was defined as more than moderately severe on a 4-point ordinal scale (0, no nausea; 1, mild; 2, moderate; 3, severe; 4, vomiting). No protocols were changed during the study period.

### 2.4 Statistical analysis

The primary outcomes of this study were EC_50_ and EC_95_ values of remifentanil for preventing cough during emergence according to smoking history. The secondary outcomes were intraoperative changes of blood pressure and heart rate, emergence and recovery profiles including the time to extubation, incidences of respiratory complications and PONV, and sedation and pain scores at PACU.

For estimating EC_50_, which is the primary outcome, Dixon up-and-down method was used. The EC_50_ and EC_95_ values of remifentanil were also estimated using isotonic regression method. According to a previous study, elucidation of EC_50_ by Dixon up-and-down method needed at least 6 success-failure/failure-success pairs and simulation studies for this method suggested that at least 20 patients should be included for obtaining stable estimates.^[19]^ To satisfy both bases, we enrolled patients until at least 7 success-failure pairs were achieved in both groups. The EC_50_ of remifentanil was defined as the mean at the mid-point of the crossover concentration for each group (ie, success-failure). They were compared using independent *T* tests. Additionally, the Ce of remifentanil was analyzed by isotonic regression method using a pooled-adjacent-violators algorithm to interpolate the EC_50_ (83%) and EC_95_ (95%) values of remifentanil within confidence intervals (CIs).^[20]^ CIs were calculated using a bootstrapping approach. If the EC_50_ and EC_95_ estimates did not overlap at 83% CI and 95% CI, the values were considered to show statistically significant differences.^[21]^

With regard to the perioperative characteristics and secondary outcomes, the chi-squared test or Fisher exact test was used to analyze categorical variables such as ASA classification, intubation attempts (once or twice), incidences of respiratory complications during emergence and PONV, and sedation score. Continuous variables, including age, height, weight, body mass index, operation time, anesthesia time, time to extubation, concentration of sevoflurane at eye-opening, and NRS at PACU were tested for normality using Kolmogorov–Smirnov test. Subsequently, Student's *t* test was used to evaluate if the variables showed normality. If the variables did not show normality, Mann–Whitney *U* test was used. A linear mixed model was used to analyze the repeated measured variables.

Data were presented as mean ± standard deviation (SD), median (interquartile range), or number (frequency). A *P*-value of <0.05 was considered statistically significant. The SPSS package (version 25.0, IBM Corporation, Armonk, NY, USA) along with R (version 3.2.5, The R foundation for Statistical Computing; https://www.r-project.org) was used for statistical analyses.

## 3 Results

**Figure 1:**
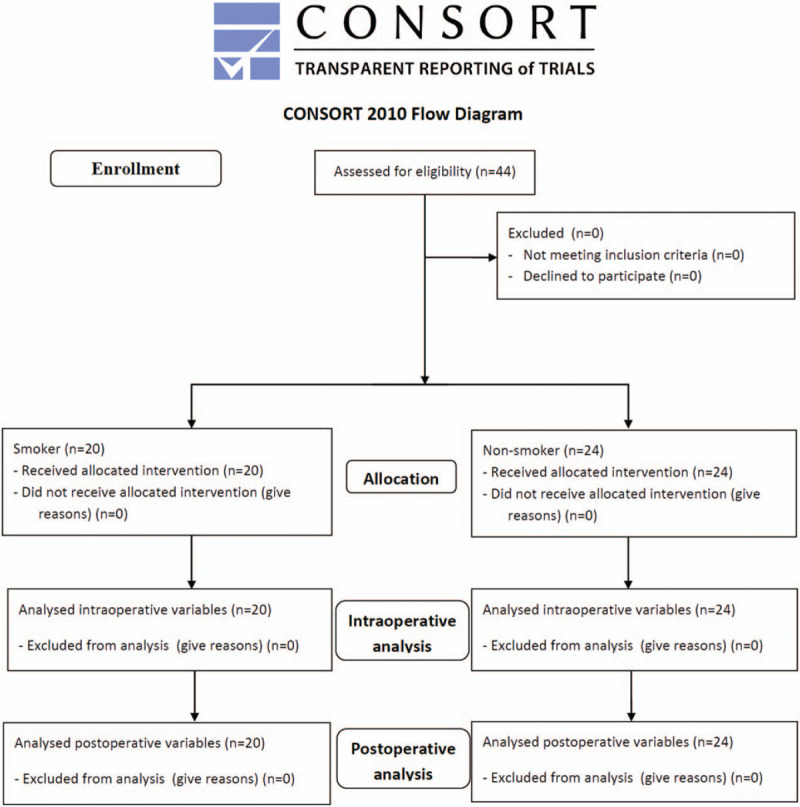
Flow diagram showing the enrollment of patients.

By Dixon up-and-down method, the patients were allocated and enrolled until the study population comprised 6 or more success-failure pairs and 20 or more patients per group. Forty-four patients were assessed with 7 success-failure pairs in each group. All the patients (20 smokers and 24 non-smokers) remained enrolled until the final analysis without withdrawal (Figure 1). The median (25^th^–75^th^ interquartile range) smoking history of smokers was 17.5 (8.1–20.0) pack × year. Patient and operation characteristics were not significantly different between the two groups (Table 1).

**Table 1:** Patients characteristics and intraoperative details.

	Smoker group (N = 20)	Non-smoker group (N = 24)
Age (years)	42.3 ± 8.0	44.5 ± 10.6
Height (cm)	174.6 ± 8.2	172.8 ± 3.9
Weight (kg)	77.6 ± 9.2	72.6 ± 8.2
Body mass index (kg/m^2^)	25.0 ± 2.7	24.5 ± 2.4
ASA classification 1/2 (n)	13/7	17/7
Intubation attempts once/twice (n)	18/2	24/0
Operation time (min)	45 (35–63)	40 (30–45)
Anesthesia time (min)	80 (73–103)	75 (68–90)

Values are mean ± standard deviation, median (25th–75th interquartile range), or number. ASA = American Society of anesthesiologist.

**Figure 2:**
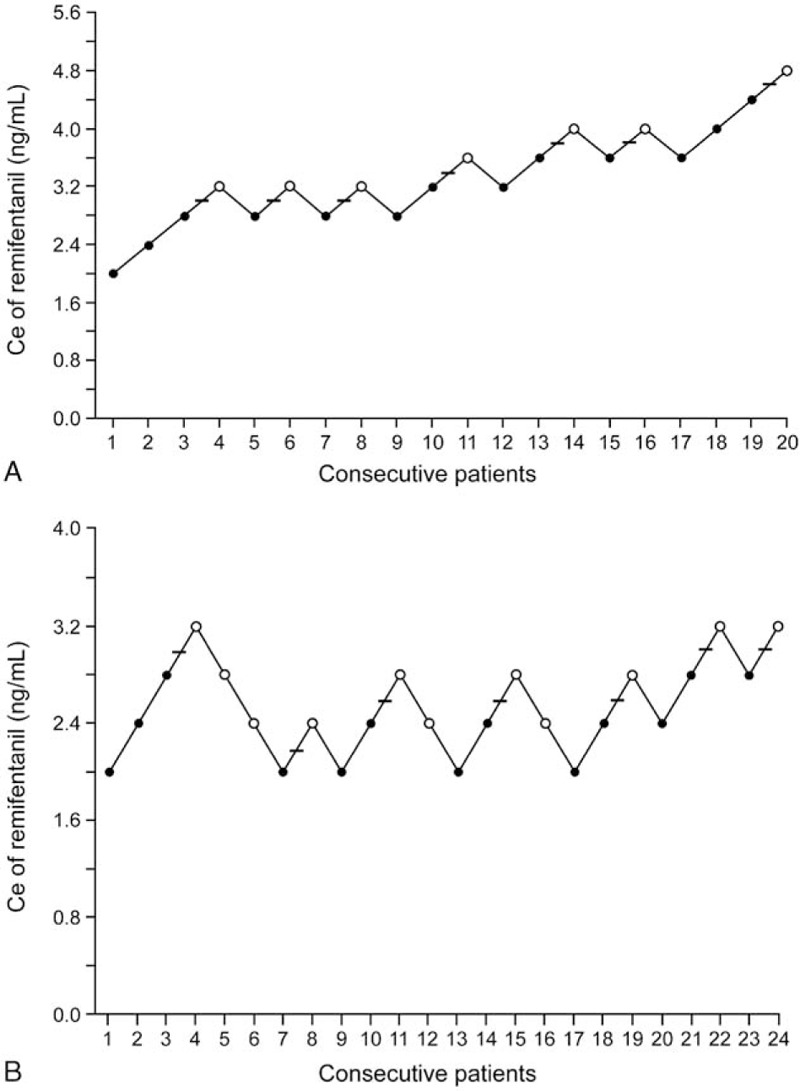
Sequences of Ce of remifentanil required for the prevention of cough during anesthetic emergence by Dixon up-and-down methods in (A) smokers and (B) non-smokers. The success and failure of smooth emergence are represented as open (○) and closed circles (●), respectively. Horizontal bars mean midpoints of the crossover concentrations for failure and success pair. Ce = effect-site concentration.

**Figure 3:**
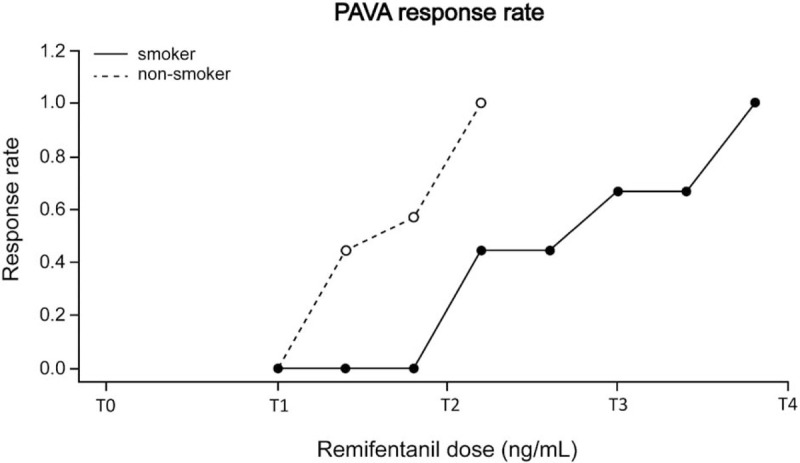
PAVA response rates in smokers (closed circle) and non-smokers (open circle). Ratio of PAVA response rate means (number of successful patients: total number of patients who were administered each Ce of remifentanil in each group). PAVA = a pooled-adjacent-violators algorithm.

**Figure 4:**
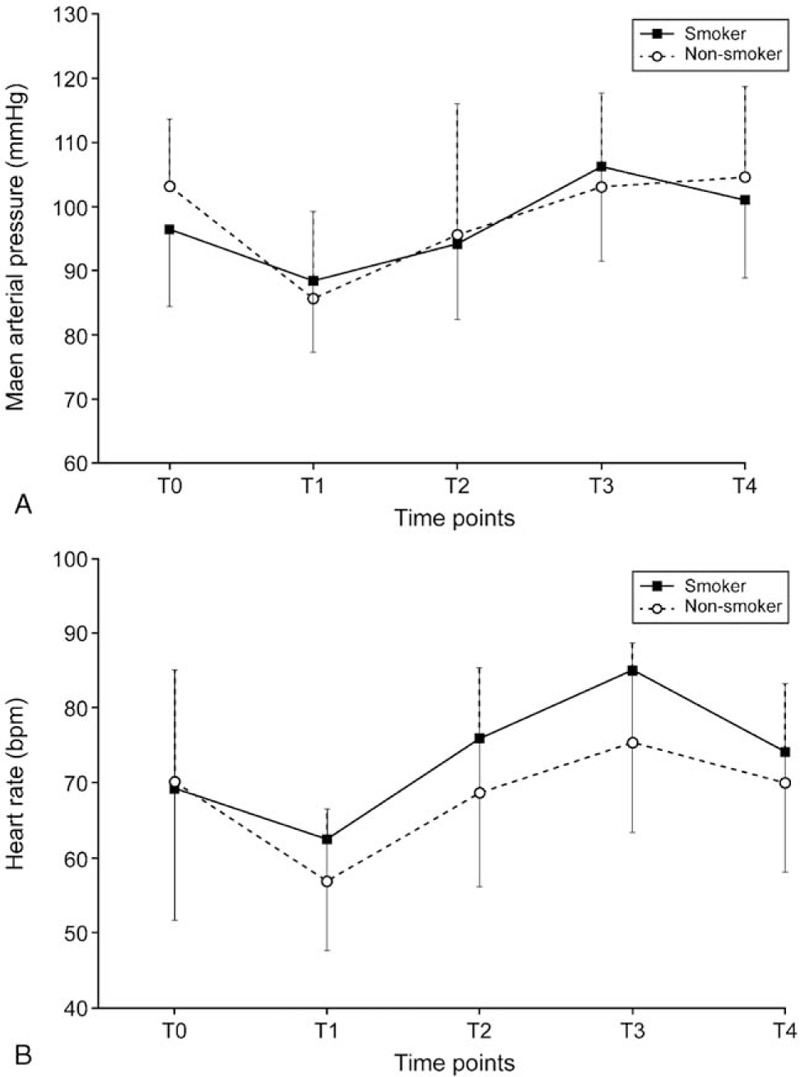
(A) Mean arterial pressure and (B) heart rate during perioperative period. Data are expressed as mean ± standard deviation. T0, before induction; T1, end of operation; T2, immediately before extubation; T3, immediately after extubation; T4, 5 min after extubation.

The success and failure of smooth emergence are presented in Figure 2. The isotonic regression curve of the probability of no cough is displayed in Figure 3. The EC_50_ and EC_95_ values of the Ce of remifentanil were calculated using Dixon method and isotonic regression method (Table 2). The EC_50_ values elucidated by Dixon method were significantly higher in smokers (3.51 ± 0.60 ng/mL) than in non-smokers (2.71 ± 0.30 ng/mL) (*P* < 0.001). The EC_50_ and EC_95_ values elucidated by isotonic regression method were 4.40 (83% CI, 4.17–4.58) ng/mL and 4.76 (95% CI, 4.73–4.78) ng/mL, respectively, in smokers and 2.58 (83% CI, 2.31–2.87) ng/mL and 3.15 (95% CI, 3.04–3.18) ng/mL, respectively, in non-smokers. Because the EC_50_ and EC_95_ values did not overlap at 83% and 95% levels of CI, respectively, the Ce of remifentanil required for preventing cough during emergence was significantly higher in smokers than in non-smokers. Intraoperative MAP and HR are presented in Figure 4. In the analysis from a linear mixed model between time and group, MAP and HR were comparable over time between the two groups throughout the perioperative period (*P* = 0.588 and *P* = 0.263, respectively).

**Table 2:** Effect-site concentration of remifentanil for suppressing emergence cough following extubation.

	Smoker group (n = 20)	Non-smoker group (n = 24)
Dixon's method		
EC_50_ of remifentanil Ce (ng/mL)	3.51 ± 0.60	2.71 ± 0.30
Isotonic regression method		
EC_50_ of remifentanil Ce (ng/mL)	4.40 (4.17–4.58)	2.58 (2.31–2.87)
EC_95_ of remifentanil Ce (ng/mL)	4.76 (4.73–4.78)	3.15 (3.04–3.18)

Values are mean ± standard deviation determined by Dixon method and the EC_50_ (83% CI) and EC_95_ (95% CI) determined by the isotonic regression method. Ce = effect-site concentration, CI = confidence interval.

The emergence and recovery outcomes are shown in Table 3. The time to extubation was shorter in non-smokers than in smokers (*P* = 0.048). Concentration of sevoflurane at the time of eye-opening was comparable between the two groups. There were two patients in each group who showed bradypnea 5 min after extubation. Bradypnea was observed at Ce (remifentanil) values of 3.6 and 4.4 ng/mL in smokers and 2.4 and 4.8 ng/mL in non-smokers. However, all 4 patients who showed bradypnea responded when they were encouraged to breathe, and none of them showed laryngospasm or desaturation. In the PACU, all the patients showed a light sedation depth responding to verbal commands (sedation score ≤3).

**Table 3:** Emergence and recovery outcomes.

	Smoker (n = 20)	Non-smoker (n = 24)	*P* value
During emergence			
Time to extubation (min)	6.6 (4.8–8.7)	5.2 (4.4–5.9)	0.048
Concentration of sevoflurane at eye opening (%)	0.20 (0.20–0.30)	0.20 (0.10–0.28)	0.294
Respiratory complications, n (%)			>0.999
Bradypnea	2 (10)	2 (8)	
Laryngospasm	0	0	
Desaturation	0	0	
At post-anesthesia care unit			
Sedation score 1/2/3, n	0/18/2	0/24/0	0.223
PONV, n (%)	2 (10)	1 (4)	0.583
Numerical rating scale	3.5 (2.0–4.8)	4.0 (3.0–5.8)	0.173

Values are mean ± standard deviation, median (25th–75th interquartile range), or number (%). Sedation score was recorded by Ramsay Sedation Scale with a range from 1 to 6. PONV = postoperative nausea and vomiting.

## 4 Discussion

In this study, we investigated the Ce of remifentanil required for preventing cough during emergence from general anesthesia in male patients according to smoking history. We found that EC_50_ and EC_95_ values were significantly higher in smokers than in non-smokers by the Dixon up-and-down method (EC_50_: 3.51 ± 0.60 ng/mL in smokers vs 2.71 ± 0.30 ng/mL in non-smokers, *P* < 0.001) and isotonic regression method [EC_50_ and EC_95_ in smokers: 4.40 (83% CI, 4.17–4.58) and 4.76 (95% CI, 4.73–4.78), vs EC_50_ and EC_95_ in non-smokers: 2.58 (83% CI, 2.31–2.87) and 3.15 (95% CI, 3.04–3.18)]. Time to extubation was longer in smokers compared to non-smokers. Respiratory complications associated with opioid infusion and the recovery profiles of the patients did not differ between smokers and non-smokers.

Emergence from general anesthesia is the time that requires the most attention to the anesthesiologists because anesthesiologists have experienced problems during emergence such as cough, inadequate ventilation, laryngospasm, and aspiration. Indeed, the incidence of such problems probably exceeds those relating to tracheal intubation.^[22]^ Of these, cough is the most common respiratory problem during emergence in the presence of a tracheal tube.^[3,23]^ Cough during emergence leads to several problems such as hemodynamic disturbances, sudden increase of intra-cavitary pressures, and negative pressure pulmonary edema.^[1]^ Since these problems can cause serious complications in patients with co-existing disease, anesthesiologists have been interested in preventing coughing during emergence.

Remifentanil infusion has been introduced as an effective method to prevent cough during emergence^[3]^ because it has a unique property of rapid onset and offset without accumulation.^[24]^ Despite many advantages of remifentanil, its infusion may lead to dose-dependent vagotonic and sympatholytic effects and opioid-related complications such as delayed emergence, respiratory depression, nausea, and vomiting.^[24]^ Hence, it is important to evaluate the optimal Ce of remifentanil that is associated with minimal complications. To date, numerous studies evaluated the optimal Ce of remifentanil according to age, sex, and type of anesthetic compound used.^[5–13]^ Smoking history is an important factor that affects cough reflex.^[25]^ However, there are no studies that evaluated the Ce of remifentanil according to smoking history during anesthetic emergence until now. Therefore, we evaluated the Ce of remifentanil according to smoking history in young and middle-aged (age range, 20–65 years) male patients considering the influence of age and sex.

Regarding sensitivity to cough reflex in smokers, the results were conflicting. Several studies revealed that smokers had a decreased sensitivity to cough reflex,^[25–27]^ while a study demonstrated that smokers had an increased sensitivity to cough reflex.^[28]^ Meanwhile, some studies revealed that the cough reflex changed with the duration of smoking cessation,^[29,30]^ and the sensitivity to cough reflex increases as early as 2 weeks after cessation of smoking.^[30]^ Generally, patients who undergo scheduled surgery are advised by clinicians to cease smoking. The surgeon in our hospital advised smoking patients to quit smoking about 2 weeks before surgery. In our study, patients who had a history of smoking at least 5 cigarettes a day for more than 6 months within the period before surgery were considered as smokers, even if they ceased smoking just before surgery following the surgeon's advice. Therefore, increased sensitivity to cough reflex by preoperative 2 weeks of smoking cessation in smokers could be the reason for higher EC_50_ and EC_95_ values in smokers than in non-smokers in our study.

Our findings are consistent with the results of previous studies that the frequency and severity of cough at the time of extubation after surgery were significantly higher in smokers than in non-smokers.^[14,15]^ Hans et al's study showed that smokers were at increased risk of coughing, independently the type of anesthetics (propofol or sevoflurane) in patients undergoing cervical spine surgery.^[14]^ Similarly, McKay et al’ study reported that smoking increased the risk of respiratory complications including coughing regardless of the choice of anesthetics (desflurane or sevoflurane).^[15]^ The new knowledge from our study is that we estimated the Ce of remifentanil to prevent cough during anesthetic emergence in smokers and nonsmokers and higher dose of remifentanil was required in smokers than in non-smokers. In clinical practice, a higher dose of remifentanil should be considered to prevent cough during anesthetic emergence in smokers than non-smokers.

There have been several studies to evaluate the relationship between smoking and the effects of opioids.^[31–34]^ In these studies, smokers seem to need higher doses of opioids to achieve the same effect. The studies revealed that smokers required greater opioids than non-smokers to control postoperative pain.^[31,32]^ In addition, the incidence of side effects of morphine such as sedation and PONV was affected by smoking history.^[33]^ A high opioids requirement in smokers probably explained an induction of enzyme CYP1A2 and increased the metabolism of drugs.^[34,35]^ However, the remifentanil has unique properties that are different from other opioids, it seems to be difficult to explain the results of our study. Remifentanil is minimally altered by hepatic function because it is eliminated by non-specific blood and tissue esterases.^[2]^

Clinicians are more interested in EC_95_ than EC_50_ for obtaining definite effects in high-risk patients. In this study, the EC_95_s of remifentanil for preventing cough were 4.76 (95% CI, 4.73–4.78) in smokers and 3.15 (95% CI, 3.04–3.18) in non-smokers. The relatively high EC_95_ of remifentanil in smokers may make the clinician hesitant to infuse remifentanil during emergence in clinical practice due to dose-dependent complications of remifentanil such as cardiovascular depression, respiratory depression, and delayed awakening. In this study, time to extubation was significantly longer in smokers [6.6 (4.8–8.7) min] than in non-smokers [5.2 (4.4–5.9) min], although hemodynamics and incidence of bradypnea during emergence were no difference between 2 groups. In actual clinical practice, combined use of other interventions such as propofol or lidocaine infusion along with remifentanil seems to help for preventing cough during emergence in high-risk patients.^[23]^

There were several limitations to this study. First, a double-blinded design was not used in this study. The outcome assessors in the operation room knew the predetermined Ce of remifentanil; this may have affected in-room outcomes. Second, although Pace et al recommended studies having at least 20 patients in the use of up-and-down method,^[19]^ the sample size was relatively small. Therefore, the small sample size could be less reliable to estimate the EC_50_ and EC_95_ values of remifentanil by isotonic regression method and to compare secondary outcomes between 2 groups. With regard to isotonic regression, because CIs are more reliable when the size of a sample is large, the EC_50_ and EC_95_ values of remifentanil should be interpreted cautiously.^[19]^ Third, the values of EC_50_ depend on clinical circumstances and which pairs we chose (eg, success-failure pairs or failure-success pairs). Therefore, the EC_50_ by Dixon up and down method is relative, not absolute values. The clinicians need to consider several factors such as age, sex, the type of surgery, and the type of anesthetic compound used, as well as the smoking history when determining the Ce of remifentanil to prevent cough during anesthetic emergence. Fourth, because cough sensitivity may vary by race, this study may be difficult to generalize to other races other than Asians.

In conclusion, the Ce of remifentanil required to prevent cough during anesthetic emergence was significantly higher in smokers than in non-smokers. Therefore, a higher dose of remifentanil should be needed to prevent cough during anesthetic emergence in smokers than in non-smokers.

## Author contributions

**Conceptualization:** Sook Young Lee.

**Data curation:** Ha Yeon Kim, Jong Bum Choi.

**Formal analysis:** Eunyoung A. Lee.

**Investigation:** Sei Hyuk Kwon.

**Methodology:** Ji Eun Kim, Sook Young Lee.

**Supervision:** Sook Young Lee.

**Writing – original draft:** Ha Yeon Kim.
